# Acupuncture Therapy for Sudden Sensorineural Hearing Loss: A Systematic Review and Meta-Analysis of Randomized Controlled Trials

**DOI:** 10.1371/journal.pone.0125240

**Published:** 2015-04-28

**Authors:** Xin-chang Zhang, Xiu-ping Xu, Wen-tao Xu, Wen-zhen Hou, Ying-ying Cheng, Chang-xi Li, Guang-xia Ni

**Affiliations:** 1 Department of Pain Management, Subei People’s Hospital of Jiangsu Province & Clinical Medical School, Yang Zhou University, Yangzhou 225001, P.R. China; 2 The Second School Medical College, Nanjing University of Chinese Medicine, Nanjing 210029, P.R. China; 3 The Second Chinese Medicine Hospital of Jiangsu Province, Nanjing 210017, P.R. China; 4 Department of Critical Care Medicine, Nanjing Zhong-da Hospital, School of Medicine, Southeast University, Nanjing 210009, P.R. China; Shanghai Jiao Tong University School of Medicine, CHINA

## Abstract

**Objective:**

Acupuncture has commonly been used in China, either alone or in combination with Western medicine, to treat sudden sensorineural hearing loss (SSHL). The purpose of this systematic review is to assess the efficacy and safety of acupuncture therapy for patients with SSHL.

**Methods:**

We searched PubMed, the Cochrane Library, Embase, China National Knowledge Internet (CNKI), Database for Chinese Technical Periodicals (VIP), and Chinese Biomedical literature service system (SinoMed) to collect randomized controlled trials of acupuncture for SSHL published before July 2014. A meta-analysis was conducted according to the Cochrane systematic review method using RevMan 5.2 software. The evidence level for each outcome was assessed using the GRADE methodology.

**Results:**

Twelve trials involving 863 patients were included. A meta-analysis showed that the effect of manual acupuncture combined with Western medicine comprehensive treatment (WMCT) was better than WMCT alone (RR 1.33, 95%CI 1.19–1.49) and the same as the effect of electroacupuncture combined with WMCT (RR 1.33, 95%CI 1.19–1.50). One study showed a better effect of electroacupuncture than of WMCT (RR 1.34, 95%CI 1.24–1.45). For mean changes in hearing over all frequencies, the meta-analysis showed a better effect with the combination of acupuncture and WMCT than with WMCT alone (MD 10.85, 95%CI 6.84–14.86). However, the evidence levels for these interventions were low or very low due to a high risk of bias and small sample sizes in the included studies.

**Conclusion:**

There was not sufficient evidence showing that acupuncture therapy alone was beneficial for treating SSHL. However, interventions combining acupuncture with WMCT had more efficacious results in the treatment of SSHL than WMCT alone. Electroacupuncture alone might be a viable alternative treatment besides WMCT for SSHL. However, given that there were fewer eligible RCTs and limitations in the included trials, such as methodological drawbacks and small sample sizes, large-scale RCTs are required to confirm the current findings regarding acupuncture therapy for SSHL.

## Introduction

Sudden sensorineural hearing loss (SSHL) is defined as an abrupt or rapidly progressing hearing loss of at least 30 dB in at least three different contiguous frequencies according to the standard pure-tone audiogram over a 72-hour period, it was first described by De Kleyn in 1944 [1,2]. Hearing loss in SSHL patients is unilateral in most cases, with bilateral involvement reported in less than 5% [3]. In addition, estimates of the incidence of SSHL have ranged from 5 to 20 per 100,000 persons per year [4–6]; however, a recent study from Germany reported an incidence as high as 160 cases per 100,000 per year [7]. SSHL, often accompanied by tinnitus and vertigo, severely affects quality of life because of limiting the ability to communicate [2], and can lead to patients experiencing anxiety and fear.

The etiology of SSHL is uncertain in most patients. It might be associated with viral infection, vascular disturbance, intra-cochlear membrane rupture or inner ear disease [8,9]. However, for the majority of patients an etiologic factor is not identified [10]. Therefore, the treatment of SSHL has usually been empirical. Currently, the most common therapies include systemic and intratympanic steroids, antiviral agents, vasodilators, and hyperbaric oxygen, among others. However, numerous systematic reviews have indicated that there is no evidence showing that steroids, antiviral agents or vasodilators can definitely be effective in the treatment of SSHL [11–15].

Acupuncture therapy widely used in China is a significant component of traditional Chinese medicine. According to traditional Chinese medicine, acupuncture therapy functions by means of stimulating certain acupoints on the human body to activate meridians and collaterals and regulate the function of Zang-fu organs, Qi and Blood. Since ancient times, there have been reports of acupuncture being used to treat symptoms of SSHL. At present, plenty of clinical studies of acupuncture for SSHL have been published with some promising results [16–22]. However, the quality of these clinical studies is uneven. To date, no systematic reviews or meta-analyses of acupuncture for SSHL have been reported. This led us to conduct a systematic review and a meta-analysis of the use of acupuncture in treating SSHL to summarize the available evidence, appraise the evidence level, and offer suggestions for future research and treatment.

## Materials and Methods

### Search strategy

The electronic databases that were used included PubMed, the Cochrane Library, Embase, the China National Knowledge Infrastructure (CNKI), Chinese Science and Technology Periodical Database (VIP), and Chinese Biomedical Literature Database (SinoMed). These databases were searched without language restriction from their inceptions until July 2014. Search terms consisted of three groups: disease (sudden sensorineural hearing loss and other related terms); intervention (acupuncture and other related terms) and study type (randomized controlled trial and other related terms). The different search terms for the above databases are shown in S1 Table. The three groups of terms were combined and the search results were downloaded into Endnote libraries for each database. The results for all searches were combined, and the duplicates were removed. We also checked the reference lists of eligible articles obtained from additional studies.

### Inclusion Criteria

Randomized controlled trials (RCTs) with no limitation on language, blinding or publication type were included if the following criteria were met:

1. Participants: participants with sudden sensorineural hearing loss that must be diagnosed with a clear description of the diagnostic criteria
2. Intervention(s): acupuncture therapy alone or with Western Medicine Comprehensive Treatment (WMCT). Acupuncture therapy only included simple manual acupuncture and electroacupuncture without differentiating between different selections of acupoints or needle materials. Scalp acupuncture, acupoint injections, laser acupuncture, moxibustion, other special forms of acupuncture, and combinations of the above were excluded
3. Comparator(s): no treatment, sham acupuncture or WMCT (i.e., hyperbaric oxygen, Western medication). WMCT in both experimental group and control group had to be identical in the same study
4. Outcome(s) evaluation: pure-tone audiometric (PTA) change, improvements in the accompanying symptoms (tinnitus or vertigo) and adverse events.

### Exclusion Criteria

Clinical trials were excluded if they did not meet the above criteria. In addition, the following types of studies were excluded: (1) studies that included oral Chinese herbal medicine; (2) studies that compared different acupuncture techniques or selections of different acupoints to control groups; and (3) duplicate studies.

### Study Selection and Data Extraction

According to the design of the retrieval strategy, one reviewer (XCZ) conducted the searches. Two evaluators (XCZ and WTX) assessed the summaries and titles independently. Irrelevant citations were excluded. If they could not decide on incorporating the study, the full texts of the articles were obtained. Two reviewers (XCZ and WTX) independently assessed the eligibility of these articles against the inclusion and exclusion criteria. Issues were resolved by agreement after discussion with a third reviewer (XPX).

Two reviewers (XCZ and WTX) extracted data independently from each included study using a predesigned data extraction form. The data extracted included publication year, country, study type, random sequence generation, allocation concealment, blinding, demographic characteristics, sample size, diagnostic criteria, treatment and control measurements, treatment duration, outcomes, follow-up period and adverse events. When study findings were uncertain or missing, we contacted the original investigators for clarification and details. We resolved any differences in opinion through rechecking the source papers and further discussion with the third reviewer (XPX).

### Assessment of Risk of Bias

In accordance with recommendations in the Cochrane Handbook of Systematic Reviews of Interventions [23], two reviewers (XCZ and XPX) independently evaluated the methodological quality of included trials using the Cochrane risk of bias assessment tool that included the following domains: random sequence generation, allocation concealment, blinding of participants and personnel, blinding of outcome assessment, incomplete outcome data, selective reporting and other biases. For each domain, the risk of bias was rated as either “low,” “high” or “unclear” [23]. If the evaluation results were inconsistent, issues were resolved by rechecking the source papers and further discussions with the third reviewer (CXL).

### Assessment of Quality of Evidence

For every outcome, the quality of evidence was assessed using GRADE profiler version 3.6 along with the consensus of two authors (XCZ and XPX) according to the Grading of Recommendations Assessment, Development and Evaluation (GRADE) system [24]. In this system, the quality of randomized trials is initially graded as high and can be downgraded due to 1) risk of bias, 2) inconsistency, 3) indirectness, 4) imprecision, and 5) publication bias. This system divides the quality of evidence into four categories: high, moderate, low and very low [25,26].

### Statistical Analysis

The meta-analysis was performed using Review Manager (RevMan) software version 5.2, provided by the Cochrane Collaboration. The results of the GRADE evidence rating were recorded in GRADE evidence profiles using the GRADE profiler software. The risk ratio (RR) was chosen for dichotomous data (effective rate). The mean difference (MD) was used when continuous data could be converted into the same units, such as the changes in the hearing thresholds. The confidence interval (CI) was established at 95%, and P values of less than 0.05 were considered statistically significant. We used I^2^ values to assess between-study heterogeneity. If I^2^ >75%, we considered the heterogeneity to be considerable, and if it could not be explained or when the number of studies was limited, a random effects model was applied [23]. Publication bias was evaluated using a funnel plot analysis if a sufficient number of trials (≥10 trials) was found. We analyzed the specific subgroups for the following factors: manual acupuncture and electroacupuncture. The results that were not amenable to presentation in forest plots are described in the text.

## Results

### Study Selection

**Fig 1:**
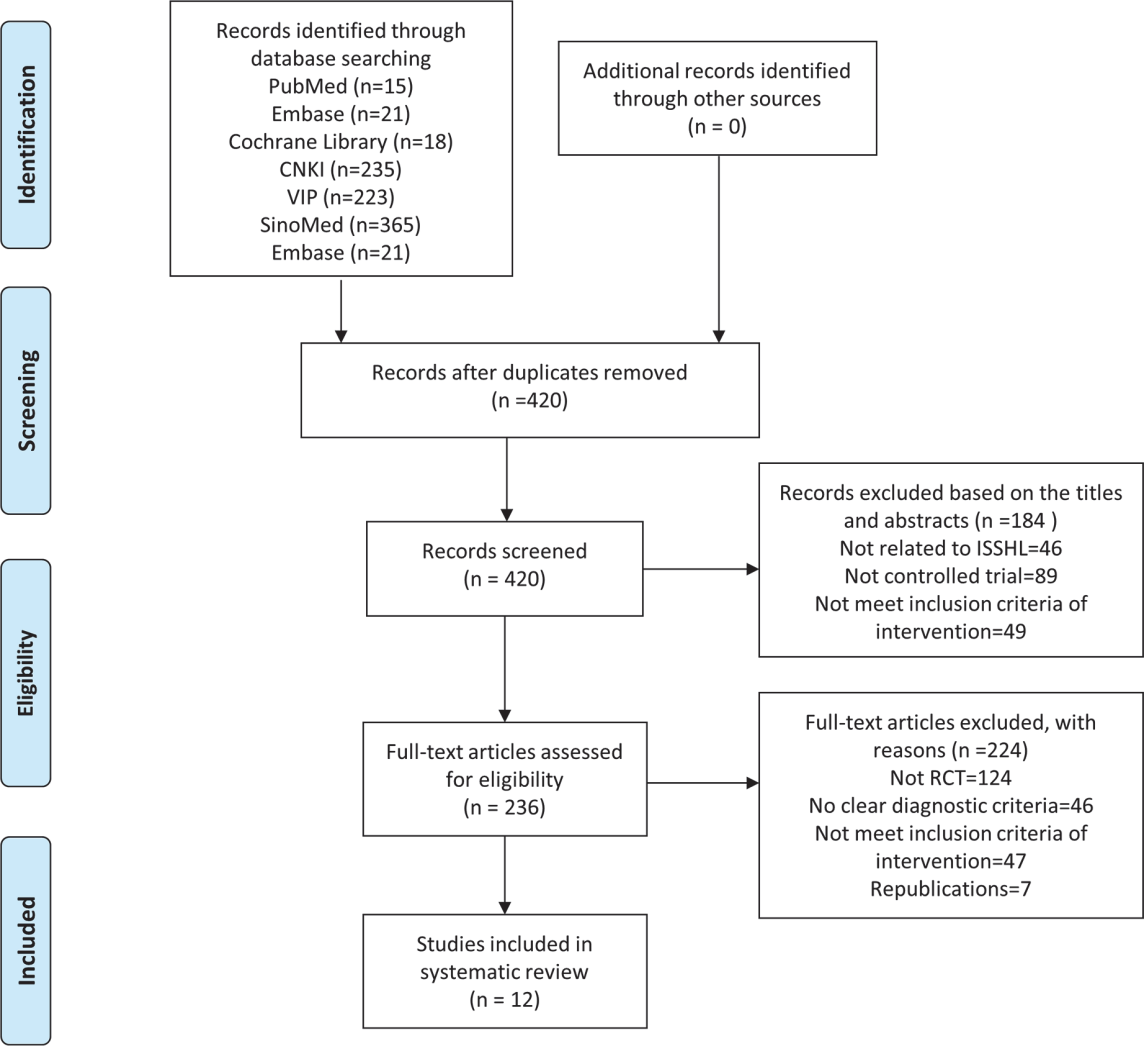
Flow diagram of the trial selection process for this systematic review.

Our initial search yielded 877 records. Of those records, 420 were retained after the duplicates were removed. After screening the titles and abstracts according to the inclusion criteria, 184 records were excluded for various reasons, such as the manuscripts being reviews, commentaries, or case reports, or not being relevant to our analysis. The full-texts of the 236 remaining articles were downloaded and assessed in detail for eligibility. After reading the full-text or contacting the authors by phone or email, a total of 224 articles were excluded because the studies were not randomized controlled trials, lacked clear diagnostic criteria, did not meet the inclusion criterion of involving an intervention, or were republications. In the end, 12 studies [27–38] were included. An overview of the study selection process is shown in Fig 1.

### Study Characteristics

All 12 trials were conducted in China. One study [32] was published in English and the remaining were in Chinese. The trials involved 863 total participants, and the sample sizes ranged from 42–130. For the diagnostic criteria of SSHL, 4 trials [32–34,37] used the 1996 Chinese Medical Association Otorhinolaryngology criteria [39]. 8 trials [27–31,35,36,38] used the 2005 Chinese Medical Association Otorhinolaryngology criteria [40]. Manual acupuncture was used in 5 studies, and electroacupuncture was used in 7 studies. Only one study [31] used acupuncture alone for the treatment group, whereas the others combined acupuncture and WMCT. The main characteristics of the included RCTs are shown in Table 1.

**Table 1:** Characteristics of included RCTs.

Study ID	Diagnostic criteria	NO.(M/F)		Interventions		Duration of treatment	Outcomes
		T	C	T (Main acupoints)	C		
Chen 2010	2005 criteria	30 (14/16)	30 (15/15)	Electroacupuncture (SJ17, SI19, GB2)+WMCT	WMCT (Low molecular dextran, ATP, Coenzyme A, Nimodipine, Gold theragran)	10 days	PTAC
Dong 2011	2005 criteria	30 (17/13)	30 (14/16)	Manual acupuncture (SI19, SJ17, GB20, LI4, SJ3, SJ5, GB43)+WMCT	WMCT (Ginkgo leaf injection, Mecobalamin, Prednisone, Hyperbaric oxygen)	14 days	PTAC, Adverse event
Huang 2014	2005 criteria	59 (33/26)	53 (29/24)	Manual acupuncture(SJ17, SJ21, GB2, SJ5, LI4, ST36, SP6, KI3, LR3, GB44)+WMCT	WMCT (Prednisone, Ginaton injection, Hyperbaric oxygen)	20 days	PTAC, Improvement of tinnitus and vertigo
Liang 2012	2005 criteria	30 (16/14)	30 (12/18)	Electroacupuncture (SJ21, SI19, GB2, SJ17, GB34, SJ5, SJ3, GB41)+WMCT	WMCT (Methylprednisolone, Alprostadil, Ganglioside, Ginaton injection, Batroxobin injection)	4 weeks	PTAC
Luo 2009	2005 criteria	30 (15/15)	30 (15/15)	Electroacupuncture (GB2, SJ17, LI4, GB43, SJ3)	WMCT (Low molecular dextran, ATP, Coenzyme A, Nimodipine, Gold theragran)	10 days	PTAC, Adverse event
Qiu 2012	1996 criteria	28 (6/22)	30 (10/20)	Electroacupuncture (SJ21, SI19, GB2, SJ17, SJ3, GB43)+WMCT	WMCT (Guanxinning injection, Ciwujia injection, Vitamin B12, Hyperbaric oxygen)	20 days	PTAC, Adverse event
Wang 1998	1996 criteria	50 (32/18)	50 (40/10)	Manual acupuncture(SJ17, ST7, SJ21, SI19, GB2, SJ3)+WMCT	WMCT (Low molecular dextran, ATP, Coenzyme A, Vitamin B6)	2 weeks	PTAC
Wang 2006	1996 criteria	73 (44/29)	57 (32/25)	Electroacupuncture (GB20, Gong xue acupoint, SJ17, SJ21, SI19)+WMCT	WMCT (Alprostadil, Vitamin B1, Vitamin B12, Vitamin C, Sibelium, Kang bing du kou fu ye)	2 weeks	PTAC
Xu(1) 2013	2005 criteria	30 (19/11)	32 (20/12)	Electroacupuncture (SJ21, SI19, GB2, SJ17, LI4, SJ3, SJ5, GB43, LR3, SP6, DU20)+WMCT	WMCT (Steroid, Mecobalamin, Alprostadil, Ginkgo leaf injection, Hyperbaric oxygen)	15 days	PTAC
Xu(2) 2013	2005 criteria	30 (19/11)	30 (16/14)	Electroacupuncture (Shen guan acupoint, SI19, GB2, SJ17, GB12)+WMCT	WMCT (Dexamethasone, Xue-shuan-tong injection, Mecobalamin, Vitamin B1, Hyperbaric oxygen)	1 month	PTAC
Zhang 2009	1996 criteria	23 (14/9)	19 (11/8)	Manual acupuncture (Si zhong acupoint, tou nie acupoint, GB20, SJ17, SJ5)+WMCT	WMCT (Dexamethasone, Ginkgo-damole Injection, Sibelium, Hyperbaric oxygen)	6 weeks	PTAC
Zhang 2013	2005 criteria	29 (11/18)	30 (16/14)	Manual acupuncture (SJ21, SJ19, SJ17,GB2, SJ3, GB43)+ WMCT	WMCT (Xue-shuan-tong injection, Honghua injection,	2 weeks	PTAC

**Abbreviations:** T, Treatment group; C, Control group; PTAC, Pure-tone audiometric change; WMCT, Western Medicine Comprehensive Treatment; 1996 criteria, 1996 Chinese Medical Association Otorhinolaryngology criteria; 2005 criteria, 2005 Chinese Medical Association Otorhinolaryngology criteria.

### Risk of Bias

**Fig 2:**
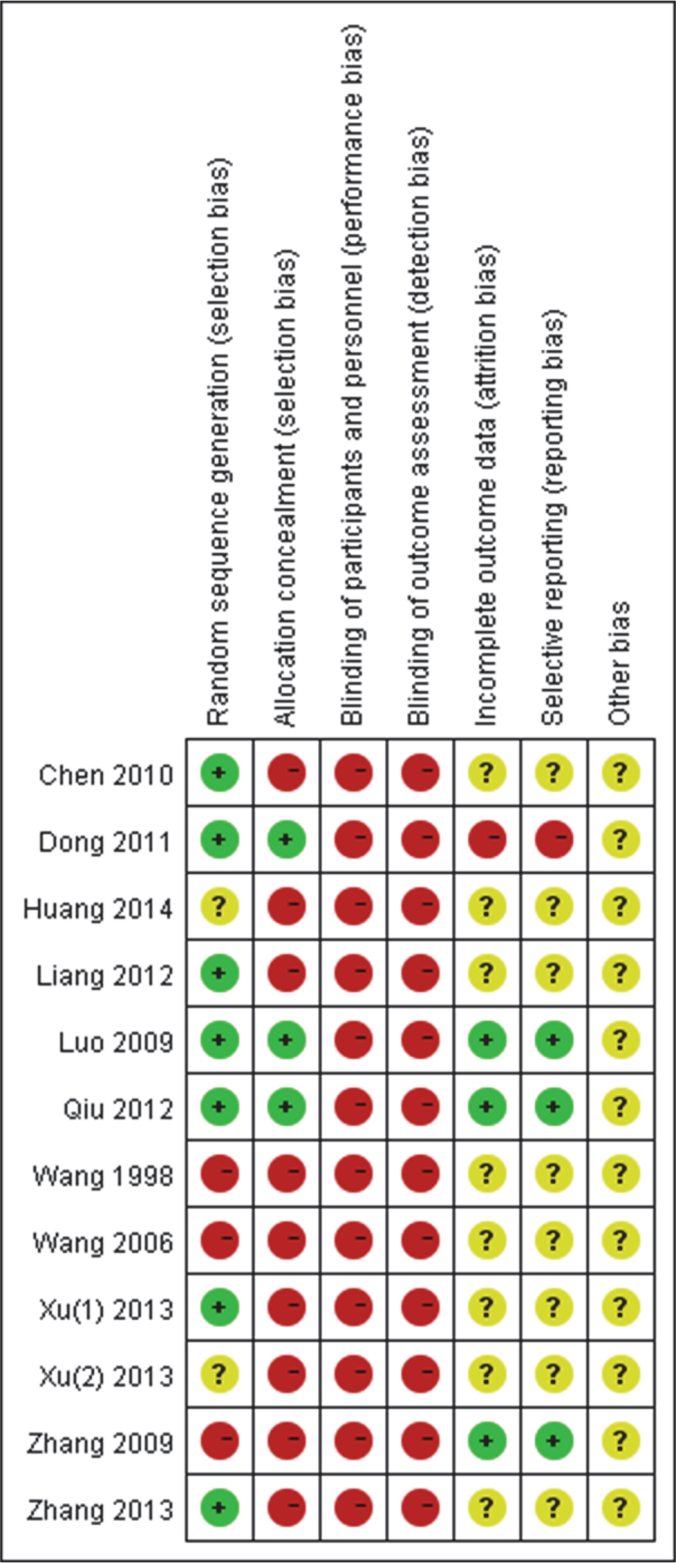
Risk of bias for each individual RCT. Red (-): high risk of bias; Yellow (?): unclear risk; Green (+): low risk of bias.

In most of the studies, the methodological information was incomplete, so we attempted to contact the authors by phone or email for more details. The patients in all 12 studies were randomly assigned to treatment or control groups. However, the exact method used for randomizing patients and allocation concealment was not clearly stated in most of studies. Only 10 trials described the methods for random sequence generation. Those methods included computer software [27,30,31], a random number table [28,32,35], visiting sequence [33,34,37] and coin toss [38]. Three studies [28,31,32] employed allocation concealment and none of the studies implemented blinding. The risk of bias for reporting participant dropouts or withdrawals and selective outcomes was low risk in 3 studies [31,32,37]. The risk of bias evaluation is presented in Fig 2.

### Effects of the Interventions

Because the intervention measures in the included studies differed, we performed subgroup analyses according to manual acupuncture and electroacupuncture for the included studies. For the control groups, the studies applied WMCT that included western medications or hyperbaric oxygen therapy.

#### Proportion of Participants with Absolute Improvement in PTA ≥15 dB

**Fig 3:**
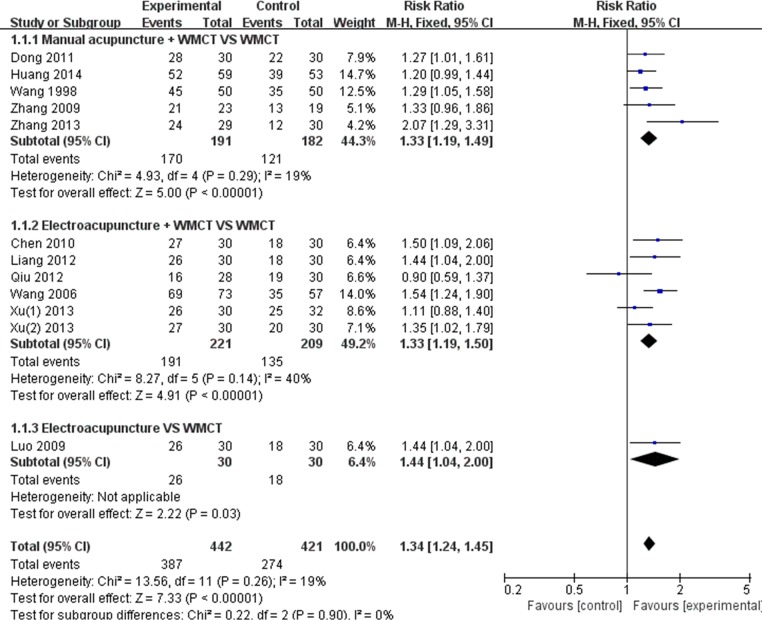
Forest plot of dichotomous data outcomes: proportion of participants with absolute improvement in PTA ≥15 dB. WMCT: western medicine comprehensive treatment.

The proportion of participants with absolute improvements in PTA ≥15 dB was reported in all studies. Five studies [28,29,33,37,38] used manual acupuncture combined with WMCT, and six studies [27,30,32,34–36] used electroacupuncture combined with WMCT. The meta-analysis showed that the effect of manual acupuncture combined with WMCT was better than WMCT alone (RR 1.33, 95%CI 1.19–1.49; *P*<0.00001; Fig 3). Furthermore, the results demonstrated better effects from the combination of electroacupuncture and WMCT than from WMCT alone (RR 1.33, 95%CI 1.19–1.50; *P*<0.00001; Fig 3). Of note, one study[31] showed a better effect of electroacupuncture than of WMCT (RR 1.44, 95%CI 1.04–2.00; *P* = 0.03; Fig 3).

#### Mean Change in Hearing over all Frequencies (dB)

**Fig 4:**
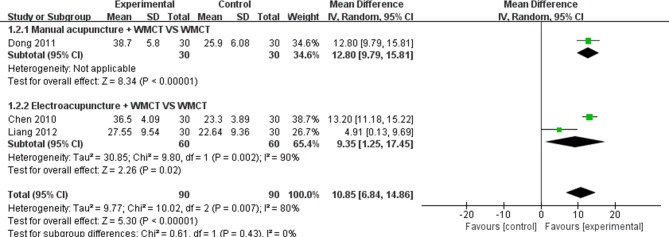
Forest plot of continuous data outcomes: mean change in hearing over all frequencies (dB). WMCT: western medicine comprehensive treatment.

Three studies [27,28,30] reported mean changes in hearing over all frequencies (dB). One study [28] used manual acupuncture combined with WMCT and the results showed that manual acupuncture combined with WMCT had a better effect than WMCT alone (MD 12.8, 95%CI 9.79–15.81; *P*<0.00001) for improving pure tone thresholds in SSHL patients. Two studies [27,30] used electroacupuncture combined with WMCT. The results indicated that electroacupuncture combined with WMCT had a better effect than WMCT alone for improving SSHL patients’ pure tone thresholds (MD 9.35, 95%CI 1.25–17.45; *P* = 0.02). In brief, the meta-analysis showed a better effect from the combination of acupuncture and WMCT than with WMCT alone (MD 10.85, 95%CI 6.84–14.86); *P*<0.00001; Fig 4) in all included studies.

#### Improvement of Tinnitus and Vertigo

Only one study [29] compared the improvement of tinnitus and vertigo between manual acupuncture combined with WMCT and WMCT alone. No significant difference was found between manual acupuncture combined with WMCT and WMCT alone in improving tinnitus and vertigo.

#### Adverse Events

Three studies [28,31,32] mentioned adverse events, of which one [28] stated that one patient had a local skin infection after repeated acupuncture. Two studies [31,32] reported no adverse events. The remaining studies were unclear about whether the patients experienced adverse effects.

### Publication Bias

**Fig 5:**
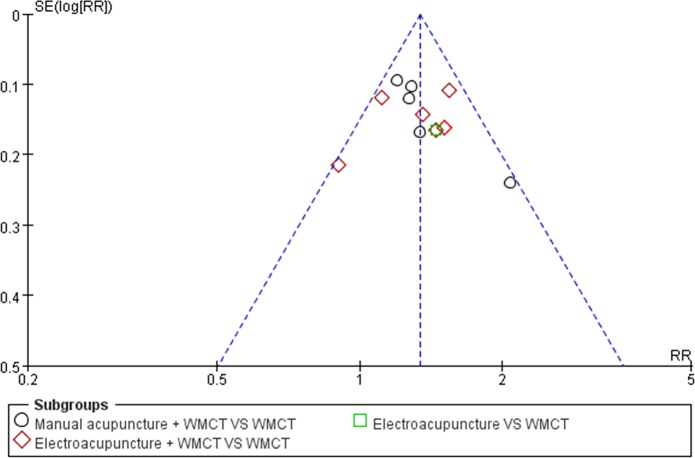
Funnel plot of the comparison between acupuncture and WMCT for the outcome of those participants with absolute improvements in PTA ≥15 dB. WMCT: western medicine comprehensive treatment.

The funnel plot for the proportion of participants with an absolute improvement in PTA ≥15 dB was created using RevMan (Fig 5). All of the included studies were from China and had small sample sizes. Although the funnel plot seemed to be symmetric, we still considered that there was potential publication bias.

### Quality of Evidence

The quality of evidence for outcome measures according to the GRADE system is presented in Table 2.

**Table 2:** GRADE evidence profile for the studies in the meta-analysis.

Quality assessment							Quality of evidence
No of studies	Design	Risk of bias	Inconsistency	Indirectness	Imprecision	Other considerations	
**Outcome: Proportion of participants with absolute improvement in PTA ≥15 dB—Acupuncture + WMCT VS WMCT**							
11	randomized trials	serious^1^	no serious inconsistency	no serious indirectness	no serious imprecision	reporting bias^7^	⊕⊕ΟΟLOW
**Outcome: Proportion of participants with absolute improvement in PTA ≥15 dB—Manual acupuncture + WMCT VS WMCT**							
5	randomized trials	serious^1^	no serious inconsistency	no serious indirectness	serious^2^	reporting bias^7^	⊕ΟΟΟVERY LOW
**Outcome: Proportion of participants with absolute improvement in PTA ≥15 dB—Electroacupuncture + WMCT VS WMCT**							
6	randomized trials	serious^1^	no serious inconsistency	no serious indirectness	serious^2^	reporting bias^7^	⊕ΟΟΟVERY LOW
**Outcome: Proportion of participants with absolute improvement in PTA ≥15 dB—Electroacupuncture VS WMCT**							
1	randomized trials	serious^3^	no serious inconsistency	no serious indirectness	serious^2^	reporting bias^4^	⊕ΟΟΟVERY LOW
**Outcome: Mean change in hearing over all frequencies (dB)—Acupuncture + WMCT VS WMCT**							
3	randomized trials	serious^1^	serious^5^	no serious indirectness	serious^2^	reporting bias^7^	⊕ΟΟΟVERY LOW
**Outcome: Mean change in hearing over all frequencies (dB)—Manual acupuncture + WMCT VS WMCT**							
1	randomized trials	serious^3^	no serious inconsistency	no serious indirectness	serious^2^	reporting bias^4^	⊕ΟΟΟVERY LOW
**Outcome: Mean change in hearing over all frequencies (dB)—Electroacupuncture + WMCT VS WMCT**							
2	randomized trials	serious^1^	serious^6^	no serious indirectness	serious^2^	reporting bias^4^	⊕ΟΟΟVERY LOW

^1^Some studies had a high risk of bias due to their methodology;

^2^Total number of events is less than 300;

^3^The study had performance bias and detection bias;

^4^Only one study or two studies;

^5^I^2^ = 80%, considerable heterogeneity;

^6^I^2^ = 90%, considerable heterogeneity;

^7^All studies were from China;

WMCT: Western Medicine Comprehensive Treatment

## Discussion

### Overview of Findings

To the best of our knowledge, this is the first meta-analysis of acupuncture therapy for SSHL patients. We included 12 studies in our review, one of which [31] compared acupuncture therapy with WMCT. The results showed that electroacupuncture was more effective than WMCT in improving patients’ hearing. However, the data were extracted from one study with a small sample size, and the study did not perform blinding of its participants or personnel or blinding of the outcome assessment. Meanwhile, the quality of evidence for this outcome was very low according to the GRADE methodology. There were 11 studies [27–30,32–38] of acupuncture with WMCT versus WMCT alone for patients with SSHL. The meta-analysis found that the treatment combining acupuncture with WMCT was more effective at improving patients’ hearing than WMCT used in the control group. The quality of evidence for this outcome was low. Compared with WMCT alone, there was a relative decrease in the mean change in hearing over all frequencies in the acupuncture plus WMCT group. In the pooled data, the mean decrease was 10.85 dB, but there were only 3 studies and considerable heterogeneity in the analysis. The quality of evidence for this outcome was very low.

SSHL is often accompanied by tinnitus. In our review one study assessed the improvement of tinnitus, and the result showed that no significant difference was found between manual acupuncture combined with WMCT and WMCT alone in improving tinnitus. This research conclusion was consistent with one previous systematic review [41]. That review included 9 RCTs and assessed the effectiveness of acupuncture for treating tinnitus. However, there was no sufficient evidences to draw definitive conclusions. Long et al [42] conducted a meta-analysis of randomized, non-randomized and observational studies on the effectiveness of acupuncture in treating Ménière’s syndrome. The results suggested a potential benefit of acupuncture for persons with Ménière’s syndrome. The symptoms of Meniere's syndrome may include hearing loss, but SSHL is different from Ménière’s syndrome because it mainly emphasizes hearing loss. In the therapeutic evaluation of Ménière’s syndrome, few studies observe an effect on hearing loss. Therefore, we conducted this study to assess the effectiveness of acupuncture for SSHL and rated the quality of evidence using the GRADE methodology.

This systematic review showed that electroacupuncture or acupuncture plus WMCT was more effective for SSHL than WMCT alone. However, the results should be interpreted with caution for several reasons. There were only 12 studies included in our research, and they all were small sample size. Most of the studies had a high risk of bias. Moreover, blinding was not performed in any of the studies. Only 10 trials described their methods for random sequence generation and only 3 studies employed allocation concealment. Meanwhile, the quality of evidence for the outcome measurements were low for one outcome, and very low for 6 outcomes according to the GRADE system. In addition, acupoints, acupuncture methods and courses of treatment varied. For example, in the control group, 6 studies used hyperbaric oxygen plus western medication, and 6 studies used western medication only. Furthermore, the specific choice of western medication varied. Taken together, these factors lead to decreased reliability of the results.

### Possible Rationale of Acupuncture Therapy for SSHL

According to traditional Chinese medicine theory, SSHL is caused by wind-cold, wind-heat, hyperactivity of liver-fire, yin deficiency and yang excess, qi stagnation and blood stasis, and stagnation of phlegm-fire [43]. If SSHL could benefit from acupuncture therapy, a possible rationale might be that acupuncture can improve local blood circulation and promote blood flow to the ear [31]. Modern medical research suggests that acupuncture performed by stimulating acupoints could improve blood circulation and blood flow in the ear and increase the oxygen supply of the ear, which are important factors in promoting auditory recovery [44]. It has also been demonstrated that acupuncture could improve symptoms, especially hearing loss, and relieve hypercoagulability. Additionally, it has been suggested that acupuncture can decrease blood viscosity, which might be one of the mechanisms that acupuncture could treat SSHL [45]. Other studies have found that acupuncture at acupoints around the ear could reduce blood viscosity, regulate the inflammatory response, improve lymph circulation and enhance the excitability and conductivity of the auditory nerve [19,46]. The specific mechanism of acupuncture therapy for SSHL is not clear. As a result, further research and discussion is in needed.

### Limitations and Implications

There are several limitations to this review. 1) Although we tried to establish corresponding search strategies to obtain all relevant RCTs of acupuncture therapy for SSHL, we did not perform a manual retrieval. Therefore, additional relevant studies might have been missed. 2) The number of studies included in our review was small, and each study had a relatively small sample size and high risk of bias. The evidence levels for each outcome were low or very low. 3) There was no evidence showing intermediate and long term effects of acupuncture due to a lack of follow-up assessments, which suggests that the effects of acupuncture over intermediate and long term follow-up periods are not well known.

Considering the above limitations, more rigorous multicenter, randomized clinical trials of acupuncture for SSHL with larger sample sizes should be launched to evaluate acupuncture’s efficiency and provide higher quality evidence for it. First, future trials should employ random sequence generation, allocation concealment and blinding correctly, report the process adequately, and include clear descriptions of dropout, withdrawal and adverse events. Second, intervention groups should use acupuncture alone rather than combining it with other treatments to allow for the exact effect of acupuncture to be obtained. Participants should be recruited carefully according to clear criteria and followed up with for a long time with clear outcome measurements. Third, researchers should register RCTs and adopt STRICTA standard [47] when performing clinical trials of acupuncture to improve the quality of future reports in this field.

## Conclusions

In conclusion, the evidence was insufficient to show acupuncture therapy alone was beneficial for treating SSHL. However, the interventions combining acupuncture with WMCT had a more efficacious result in the treatment of SSHL than WMCT alone. Electroacupuncture alone might be a viable alternative to WMCT for SSHL. However, given the few eligible RCTs and limitations in the included trials, such as methodological drawbacks and small sample sizes, large-scale RCTs are warranted to confirm the current findings regarding acupuncture as an intervention for SSHL.

## Supporting Information

S1 PRISMA Checklist PRISMA checklist. (DOC)

S1 Table Search terms used in databases. (DOC)

## References

[1] Liu SC, Kang BH, Lee JC, Lin YS, Huang KL, Liu DW, et al Comparison of therapeutic results in sudden sensorineural hearing loss with/without additional hyperbaric oxygen therapy: a retrospective review of 465 audiologically controlled cases. Clinical otolaryngology: official journal of ENT-UK; official journal of Netherlands Society for Oto-Rhino-Laryngology & Cervico-Facial Surgery. 2011; 36: 121–128. 10.1111/j.1749-4486.2011.02303.x . 21414179

[2] Stachler RJ, Chandrasekhar SS, Archer SM, Rosenfeld RM, Schwartz SR, Barrs DM, et al Clinical practice guideline: sudden hearing loss. Otolaryngology—head and neck surgery: official journal of American Academy of Otolaryngology-Head and Neck Surgery. 2012; 146: S1–35. 10.1177/0194599812436449 . 22383545

[3] Schreiber BE, Agrup C, Haskard DO, Luxon LM. Sudden sensorineural hearing loss. The Lancet. 2010; 375: 1203–1211. 10.1016/S0140-6736(09)62071-7 20362815

[4] Byl FM Jr. Sudden hearing loss: eight years' experience and suggested prognostic table. The Laryngoscope. 1984; 94: 647–661. . 6325838

[5] Plaza G, Durio E, Herraiz C, Rivera T, Garcia-Berrocal JR, Asociacion Madrilena de ORL. [Consensus on diagnosis and treatment of sudden hearing loss. Asociacion Madrilena de ORL]. Acta otorrinolaringologica espanola. 2011; 62: 144–157. 10.1016/j.otorri.2010.09.001 . 21112580

[6] Wu CS, Lin HC, Chao PZ. Sudden sensorineural hearing loss: evidence from Taiwan. Audiology & neuro-otology. 2006; 11: 151–156. 10.1159/000091198 . 16449805

[7] Klemm E, Deutscher A, Mosges R. [A present investigation of the epidemiology in idiopathic sudden sensorineural hearing loss]. Laryngo- rhino- otologie. 2009; 88: 524–527. 10.1055/s-0028-1128133 . 19194837

[8] Lazarini PR, Camargo AC. Idiopathic sudden sensorineural hearing loss: etiopathogenic aspects. Brazilian journal of otorhinolaryngology. 2006; 72: 554–561.. 17143437 10.1016/S1808-8694(15)31004-1 PMC9445700

[9] Suckfull M. Perspectives on the pathophysiology and treatment of sudden idiopathic sensorineural hearing loss. Deutsches Arzteblatt international. 2009; 106: 669–675; quiz 76. 10.3238/arztebl.2009.0669 . 19946432 PMC2780011

[10] Kuhn M, Heman-Ackah SE, Shaikh JA, Roehm PC. Sudden sensorineural hearing loss: a review of diagnosis, treatment, and prognosis. 2011; 15: 91–105. 10.1177/1084713811408349 . PMC4040829 21606048

[11] Crane RA, Camilon M, Nguyen S, Meyer TA. Steroids for treatment of sudden sensorineural hearing loss: A meta-analysis of randomized controlled trials. Laryngoscope. 2015; 125: 209–217. 10.1002/lary.24834 . 25045896

[12] Wei BP, Stathopoulos D, O'Leary S. Steroids for idiopathic sudden sensorineural hearing loss. The Cochrane database of systematic reviews. 2013; 7: CD003998. 10.1002/14651858.CD003998.pub3 . 23818120 PMC7390468

[13] Chau JK, Cho JJ, Fritz DK. Evidence-based practice: management of adult sensorineural hearing loss. Otolaryngologic clinics of North America. 2012; 45: 941–958. 10.1016/j.otc.2012.06.002 . 22980677

[14] Wang M, Liu Y, Du Z, Zhu X, Luo G. [A systematic review of vasodilators for sudden sensorineural hearing loss]. Lin chuang er bi yan hou tou jing wai ke za zhi = Journal of clinical otorhinolaryngology, head, and neck surgery. 2010; 24: 869–871. . 21174745

[15] Conlin AE, Parnes LS. Treatment of sudden sensorineural hearing loss: I. A systematic review. Archives of otolaryngology—head & neck surgery. 2007; 133: 573–581. 10.1001/archotol.133.6.573 . 17576908

[16] Zhang CY, Wang Y. [Comparison of therapeutic effects of deep needling and shallow needling on sudden deafness]. Zhongguo zhen jiu = Chinese acupuncture & moxibustion. 2006; 26: 256–258. Epub 2006/04/29. . 16642610

[17] Ji J, Fang XL. [Clinical observation on warming-removing obstruction needling method for treatment of sudden tinnitus and deafness]. Zhongguo zhen jiu = Chinese acupuncture & moxibustion. 2008; 28: 353–355. Epub 2008/07/26. . 18652328

[18] Zhang XZ, Wang RM, Qian J. [Observation on therapeutic effects of different treatments for sudden deafness]. Zhongguo zhen jiu = Chinese acupuncture & moxibustion. 2009; 29: 525–528. Epub 2009/10/20. . 19835117

[19] Fan XH, Ding YN, Chang XH, Ouyang YL, Xie Q. [Comparative observation on acupuncture-moxibustion and western medication for treatment of sudden deafness]. Zhongguo zhen jiu = Chinese acupuncture & moxibustion. 2010; 30: 630–632. . 20942277

[20] Yin CS, Park HJ, Nam HJ. Acupuncture for refractory cases of sudden sensorineural hearing loss. Journal of alternative and complementary medicine (New York, NY). 2010; 16: 973–978. Epub 2010/08/14. 10.1089/acm.2009.0542 . 20704516

[21] Huang N, Li C. Recurrent sudden sensorineural hearing loss in a 58-year-old woman with severe dizziness: a case report. Acupuncture in medicine: journal of the British Medical Acupuncture Society. 2012; 30: 56–59. Epub 2011/12/16. 10.1136/aim.2010.010003 . 22169707

[22] Luo CL, He TY, Qin XG, Chen Q, Tian XG, Hou CY. [Clinical observation on idiopathic sudden hearing loss treated by warming-promoting needling technique]. Zhongguo zhen jiu = Chinese acupuncture & moxibustion. 2012; 32: 981–983. Epub 2012/12/12. . 23213981

[23] Higgins JPT GSe. Cochrane Handbook for Systematic Reviews of Interventions Version 5.1.0 [updated March 2011]. The Cochrane Collaboration, 2011. Available: www.cochrane-handbook.org.

[24] Guyatt GH, Oxman AD, Vist GE, Kunz R, Falck-Ytter Y, Alonso-Coello P, et al GRADE: an emerging consensus on rating quality of evidence and strength of recommendations. Bmj. 2008; 336: 924–926. 10.1136/bmj.39489.470347.AD . 18436948 PMC2335261

[25] Balshem H, Helfand M, Schunemann HJ, Oxman AD, Kunz R, Brozek J, et al GRADE guidelines: 3. Rating the quality of evidence. Journal of clinical epidemiology. 2011; 64: 401–406. 10.1016/j.jclinepi.2010.07.015 . 21208779

[26] Schünemann H, Brożek J, Guyatt G, Oxman A, editors. GRADE handbook for grading quality of evidence and strength of recommendation Version 3.6 [updated October 2011]. The GRADE Working Group, 2011 Available: http://wwwgradeproorg/gradepro/.

[27] Chen XL, Luo RH, Xu K, Luo RH. Clinical research of electroacupuncture combined with medicine for treatment of sudden deafness. Journal of New Chinese Medicine. 2011; 42: 76–77.

[28] Dong LH, Yang XB, AN JM. 30 cases of sudden hearing loss treated mainly by Hegu needling. Journal of Shanxi College of Traditional Chinese Medicine. 2011; 34: 70–71.

[29] Huang W, Yue HY, Hu H, Yu YJ, Tan J, Hao YN, et al Combination of acupuncture and western medicine for the treatment of sudden deafness: report of 59 cases. Shanghai Journal of Traditional Chinese Medicine. 2014; 48: 50–52.

[30] Liang JG, Zhang WR, Cai WW. Clinical observation on treatment of sudden hearing loss with combination of TCM and western medicine. Zhong Yi Er Bi Hou Ke Xue Yan Jiu. 2012; 11: 36–38+18.

[31] Luo RH, Zhou J, Huang YS, Xu K. [Observation on therapeutic effect of electroacupuncture for treatment of sudden hearing loss]. Zhongguo zhen jiu = Chinese acupuncture & moxibustion. 2009; 29: 185–187. 19358498

[32] Qiu L, Zheng X, Xie F, Zhang M, Zhang J, Yuan SB, et al Clinical observation on the different frequency hearing damages in sudden deafness treated by electroacupuncture combined with western medicine comprehensive therapy. World Journal of Acupuncture—Moxibustion. 2012; 22: 22–27.

[33] Wang CH, Yuan J, Liu YR, Liao HS, Zhang ZH. Clinical study of acupuncture treatment on the sudden deafness. Acupuncture Research. 1998; 1: 5–7.

[34] Wang Y, Gao WB. Clinical study of electro-nape-acupuncture in the treatment of sudden deafness. Journal of Clinical Acupuncture and Moxibustion. 2006; 22: 33–34.

[35] Xu WY, Li T. Clinical research of electro-acupuncture assisted treatment for sudden deafness. China Journal of Chinese Medicine. 2013; 28: 1915–1917.

[36] Xu YG, Zhang J, Yang JF, Wei JZ. The curative effect observation of shen guan acupoint combined four acupoints around ear for treatment of sudden hearing loss. Journal of New Chinese Medicine. 2013; 45: 127–128.

[37] Zhang HT, Yuan T. Observation on therapeutic effect of matrix acupuncture in the treatment of sudden deafness. Gansu science and technology. 2009; 25: 150–151.

[38] Zhang B, Wang SX, Zhu HH. Clinical observation on treatment of sudden deafness with acupuncture and medication. Journal of New Chinese Medicine. 2013; 45: 97–98.

[39] Otorhinolaryngology of Chinese Medical Association, Editorial board of Chinese Journal of Otolaryngology. The diagnosis and efficacy grade of sudden hearing loss. Chinese Journal of Otolaryngology. 1997; 32: 72.

[40] Editorial board of Chinese Journal of Otorhinolaryngology Head and Neck Surgery, Otorhinolaryngology Head and Neck Surgery of Chinese Medical Association. Guideline of diagnosis and treatment of sudden hearing loss (2005, Jinan). Chinese Journal of Otorhinolaryngology Head and Neck Surgery. 2006; 41: 569.

[41] Kim JI, Choi JY, Lee DH, Choi TY, Lee MS, Ernst E. Acupuncture for the treatment of tinnitus: a systematic review of randomized clinical trials. BMC complementary and alternative medicine. 2012; 12: 97 10.1186/1472-6882-12-97 . 22805113 PMC3493359

[42] Long AF, Xing M, Morgan K, Brettle A. Exploring the Evidence Base for Acupuncture in the Treatment of Meniere's Syndrome-A Systematic Review. Evidence-based complementary and alternative medicine: eCAM 2011: 429102. 10.1093/ecam/nep047 . 19505974 PMC3136456

[43] Su CX, Yan LJ, Lewith G, Liu JP. Chinese herbal medicine for idiopathic sudden sensorineural hearing loss: a systematic review of randomised clinical trials. Clinical otolaryngology: official journal of ENT-UK; official journal of Netherlands Society for Oto-Rhino-Laryngology & Cervico-Facial Surgery. 2013; 38: 455–473. 10.1111/coa.12198 . 24209508

[44] Liu YX, Cao XM, Li H, Yu F, Li XN. Clinical and hemorheological effect of contralateral acupuncture treatment for sudden deafness. Acta Chinese Medicine and Pharmacology. 2011; 39: 111–113.

[45] Wang CH, Yang LW, Wang HC, Wang ZO, Feng W, Li T, et al Effect of acupuncture treatment on hemorheology in the patient of sudden deafness. Zhongguo Zhen Jiu. 2003; 23: 87–88.

[46] Ying HZ, Yan QF. Effects of acupuncture combined hyperbaric oxygen in treatment of sudden deafness on blood rheology and clinical efficacy. China modern doctor. 2014; 52: 13–16.

[47] MacPherson H, Altman DG, Hammerschlag R, Youping L, Taixiang W, White A, et al Revised STandards for Reporting Interventions in Clinical Trials of Acupuncture (STRICTA): extending the CONSORT statement. PLoS medicine. 2010; 7: e1000261 10.1371/journal.pmed.1000261 . 20543992 PMC2882429

