# Correction: Acupuncture Therapy for Sudden Sensorineural Hearing Loss: A Systematic Review and Meta-Analysis of Randomized Controlled Trials

**DOI:** 10.1371/journal.pone.0131031

**Published:** 2015-06-15

**Authors:** Xin-chang Zhang, Xiu-ping Xu, Wen-tao Xu, Wen-zhen Hou, Ying-ying Cheng, Chang-xi Li, Guang-xia Ni

There is an error in affiliation 2 for authors Xin-chang Zhang, Wen-tao Xu, Wen-zhen Hou, Ying-ying Cheng, and Guang-xia Ni. Affiliation 2 should be: The NO.2 Clinical Medical College, Nanjing University of Chinese Medicine, Nanjing 210023, P.R. China.

## References

[1] Zhang X-c, Xu X-p, Xu W-t, Hou W-z, Cheng Y-y, Li C-x, et al (2015) Acupuncture Therapy for Sudden Sensorineural Hearing Loss: A Systematic Review and Meta-Analysis of Randomized Controlled Trials. PLoS ONE 10(4): e0125240 doi: 10.1371/journal.pone.0125240 25919000 10.1371/journal.pone.0125240 PMC4412536

