# Supplementary material for: Acupuncture Therapy for Sudden Sensorineural Hearing Loss: A Systematic Review and Meta-Analysis of Randomized Controlled Trials
Source: PLoS One. 2015 Apr 28;10(4):e0125240. doi: 10.1371/journal.pone.0125240 (PMC4412536; doi:10.1371/journal.pone.0125240)
Supplement: S1 Table — (DOC) [file pone.0125240.s002.doc]

**Table 1．****Search terms used in databases**

| 1. PubMed |
| --- |
| #1 Search  sudden hearing loss  #2 Search sudden sensorineural hearing loss  #3 Search sudden deafness  #4 Search  Idiopathic sudden sensorineural hearing loss  #5 Search acupuncture  #6 Search acupressure  #7 Search  acupoint  #8 Search electroacupuncture  #9 Search #1 OR #2 OR #3 OR #4  #10 Search #5 OR #6 OR #7 OR #8  #11 Search (#9) AND #10 |
| 1. EMBASE |
| #1 Search sudden hearing loss  #2 Search sudden sensorineural hearing loss  #3 Search sudden deafness  #4 Search Idiopathic sudden sensorineural hearing loss  #5 Search acupuncture  #6 Search acupressure  #7 Search acupoint  #8 Search electroacupuncture  #9 Search #1 OR #2 OR #3 OR #4  #10 Search #5 OR #6 OR #7 OR #8  #11 Search (#9) AND #10 |
| 1. Cochrane Library |
| #1 Search sudden hearing loss  #2 Search sudden sensorineural hearing loss  #3 Search sudden deafness  #4 Search Idiopathic sudden sensorineural hearing loss  #5 Search acupuncture  #6 Search acupressure  #7 Search acupoint  #8 Search electroacupuncture  #9 Search #1 OR #2 OR #3 OR #4  #10 Search #5 OR #6 OR #7 OR #8  #11 Search (#9) AND #10  #12 Search #11 AND Trial |
| 1. SinoMed |
| #1 Abstract: "tu fa xing er long" OR "tu fa xing long" OR “tu long”  #2 Abstract: "zhen ci" OR "zhen" OR "ci" OR "dian zhen"  #3 #1AND#2 |
| 1. CNKI |
| #1 Search: Topic="tu fa xing er long" OR "tu fa xing long" OR “tu long”  #2 Search: Topic="zhen ci" OR "zhen" OR "ci" OR "dian zhen" #3 FT= "randomised"  #3 Search: #1AND#2 |
| 1. VIP |
| #1 Search: Title or Keywords="tu fa xing er long" OR "tu fa xing long" OR “tu long”  #2 Search: Title or Keywords="zhen ci" OR "zhen" OR "ci" OR "dian zhen"  #3 Search: #1AND#2 |
